# Evaluation of Leishmanization Using *Iranian Lizard Leishmania* Mixed With CpG-ODN as a Candidate Vaccine Against Experimental Murine Leishmaniasis

**DOI:** 10.3389/fimmu.2020.01725

**Published:** 2020-10-23

**Authors:** Nafiseh Keshavarzian, Mina Noroozbeygi, Mostafa Haji Molla Hoseini, Farshid Yeganeh

**Affiliations:** Department of Immunology, School of Medicine, Shahid Beheshti University of Medical Sciences, Tehran, Iran

**Keywords:** adjuvant, CpG, *Iranian Lizard Leishmania*, live vaccine, immunization, parasite burden

## Abstract

**Background and Objectives:** The live non-pathogenic Leishmania tarantolae has recently provided a promising approach as an effective vaccine candidate against experimental leishmaniasis (ILL). Here, we evaluated the immunoprotective potential of the live Iranian Lizard Leishmania mixed with CpG adjuvant against *L. major* infection in BALB/c mice.

**Methods:** Four groups of female BALB/c mice were included in the study. The first and second groups received PBS and CpG, respectively. The immunized groups received 2 × 10^5^ ILL promastigotes and the CpG-mixed ILL (ILL+CpG). Injections were performed subcutaneously in the right footpad. Three weeks later, all mice were challenged with 2 × 10^5^ metacyclic promastigotes of *Leishmania major*^*EGFP*^; inoculation was done in the left footpad. The measurement of footpad swelling and *in vivo* fluorescent imaging were used to evaluate disease progress during infection course. Eight weeks after challenge, all mice were sacrificed and the cytokines levels (IFN-γ, IL-4, and IL-10) and sera antibodies concentrations (IgG2a and IgG1) using ELISA assay, nitric oxide production using Griess assay, and arginase activity in cultured splenocytes, were measured. In addition, direct fluorescent microscopy analysis and qPCR assay were used to quantify the splenic parasite burden.

**Result:** The results showed that mice immunized with ILL+CpG were protected against the development of the dermal lesion. Moreover, they showed a significant reduction in the parasite load, in comparison to the control groups. The observed protection was associated with higher production of IFN-γ, as well as a reduction in IL-4 level. Additionally, the results demonstrated that arginase activity was decreased in ILL+CpG group compared to other groups.

**Conclusion:** Immunization using ILL+CpG induces a protective immunity; indicating that ILL with an appropriate adjuvant would be a suitable choice for vaccination against leishmaniasis.

## Introduction

Leishmaniasis is a disease caused by protozoan parasites of the genus *Leishmania*, which is transmitted to mammalian hosts such as humans, dogs, and mice by the bite of an infected female phlebotomine sandfly. *Leishmania* has an obligate intracellular proliferation cycle within phagocytic cells. The consequence of *Leishmania* infection is a chronic disease with diverse clinical manifestations that vary from self-limiting cutaneous leishmaniasis to fatal visceral leishmaniasis (Kala-azar) (1). More than 350 million people are at risk of leishmaniasis in 88 countries, wherein 0.7–1 million new cases occur annually; of which about 90% occurs in middle east countries (2).

Prevention methods or treatment options for leishmaniasis are limited, and each suffers from various shortcomings. Vaccination seems to be the best choice to control leishmaniasis, as patients who recuperate from the disease elicit a complete protective immunity, not only against parasite species causing the primary infection but also against other *Leishmania* species (3). However, there is currently no effective vaccine against leishmaniasis. So far, different strategies have been developed to achieve a safe and protective vaccine for leishmaniasis. Among them, leishmanization is the best way to evoke a protective durable immune response. Leishmanization was performing in the Middle East, which was based on the deliberate inoculation of live infective *Leishmania* parasites into the invisible regions of the body to induce protection against cutaneous leishmaniasis (4). This procedure has been stopped due to safety concerns (5). Therefore, the induction of protective immunity using either inactivated/attenuated or non-pathogenic live vaccines can be an important step toward controlling the disease. It is important to note that, long-term immunity against Leishmania infection needs persistent infection with a low number of parasites in the host cells (6), therefore, vaccination using live infective but non-pathogenic parasites seems to be the proper approach. Recently, leishmanization using *L. tarantolae* that is non-pathogenic to mammals has received much attention (6). Previous results revealed that although *L. tarantolae* is able to infect phagocytic cells, it cannot persist in the cells for a long time, and therefore cannot elicit a potent long-lasting immune response (6, 7). To overcome this problem, researchers have developed several recombinant *L. tarantolae* expressing a range of virulence factors of various Leishmania species (8–10). An alternative approach might be the use of a live, non-pathogenic *Leishmania* along with an adjuvant that promotes more potent immunity (11). The ability of CpG-containing immunostimulatory oligodeoxynucleotides (CpG-ODNs) to induce both innate and adaptive cellular immune responses has made them attractive choices for vaccination against intracellular pathogens (12–15). CpG-ODNs stimulate DCs for making IL-12 and IL-18 and also co-stimulatory molecules, enabling induction of a stronger T_H_1 response (16–18). CpG-ODNs also have the ability to induce cytotoxic T cells and antibody responses (14). Thus, they are a good choice for vaccination against intracellular pathogens such as *Leishmania*.

In the present study, we used *Iranian Lizard Leishmania* (*ILL*) as a live vaccine with CpG-ODNs as adjuvants against *L. major*^*EGFP*^ challenge. The results showed that immunization of BALB/c mice using live ILL mixed with CpG-ODNs induced protective immunity against *L. major* infection, which was confirmed by the absence of lesions at the site of infection and low parasitic load in the draining lymph nodes and spleens.

## Materials and Methods

### Mice and Parasites

Female BALB/c mice (6–8 weeks) were purchased from the Pasture Institute of Iran. The animal care procedures were reviewed and approved by the Institutional Animal Care and Research Advisory Committee of the Shahid Beheshti University of Medical Sciences, Tehran, Iran (IR.SBMU.MSP.REC. 1396.743).

*Iranian Lizard Leishmania* (a kind gift from Bahram Kazemi^1^) and enhanced green fluorescent protein (EGFP) expressing *L. major* (MRHO/IR/75/ER) were grown in RPMI 1640 medium (Gibco, USA), supplemented with 1% Penstrep (Gibco, USA) and 10% (V/V) heat-inactivated fetal bovine serum (FBS, Gibco, USA) at 26°C.

### Vaccine Preparation, Immunization, and Challenge Protocol

Phosphorothioate-modified ODN sequence 1826 containing two CpG motifs (underlined 5′-TCCATGACGTTCCTGACGTT-3′) (Microsynth Group, Switzerland), was reconstituted at 5,000 μg/mL in 1 mL sterile PBS and diluted to 50 μg/μL in sterile and pyrogen-free PBS.

The sample size considering 10 percent attrition was calculated according to the previously described method (19). Mice (*n* = 20) were divided into five groups. Control groups received PBS and CpG ODN (50 μg/mL). The immunized groups received 2 × 10^5^ ILL promastigotes and 2 × 10^5^ ILL plus CpG (50 μg/mL). All injections were done subcutaneously (SC) in the right footpad, in a volume of 40 μL of sterile and non-pyrogenic PBS as a diluent. Three weeks after the immunization, all mice were challenged by inoculation of 2 × 10^5^ infective-stage promastigotes (metacyclic) of *L. major*^*EGFP*^ (40 μL), subcutaneously in the left footpad. After the challenge, the footpad diameter was measured using a metric caliper twice a week. A group of healthy mice, which received no injections, was also included in the study. Eight weeks after challenge, mice were sacrificed for isolation of spleens and popliteal lymph nodes to determine the parasite burden and evaluate recall antigen-specific, cytokine production. A group of healthy mice, which received no injections, was maintained for 11 weeks to be compared among test groups.

### Parasite Quantitation by Fluorescent *in vivo* Imaging

To monitor the infection progress, one mouse from each group (to reduce the risk of any side effects of anesthetizing including stress and death) was selected randomly, and *in vivo* fluorescent imaging was performed in the 7 and 8th weeks of post-challenge, using Kodak FX Pro imaging system (Kodak Molecular Imaging Systems, USA). The uninfected (healthy) mouse was used as negative control. To reduce the fluorescence background, the skin of the legs, and feet of mice was epilated. Before imaging, mice were anesthetized with Isoflurane 2% through the inhalation route (20). Semi-quantitative fluorescent intensity was measured for each image using KODAK Molecular Image software.

### Parasite Quantitation by Direct Fluorescent Microscopy (DFM)

Eight weeks after infection with *L. major*^*EGFP*^, popliteal lymph nodes were isolated and cell suspensions were prepared in RPMI 1640 medium, and the cells infected with the *L. major*^*EGFP*^ were counted on a hemocytometer using a fluorescent microscope Infection indexes were determined by multiplying the percentage of infected cells by the average number of parasite per cell. Each individual coverslip was first Giemsa stained and then analyzed under a light microscope. To accomplish this, coverslips were divided into four areas, and using 1000X magnitude the number of infected cells was determined in 100 cells in each area. An average of four areas was used to determine the mean percent of the infected macrophages. The average number of parasites per cell was determined by counting the total number of intercellular amastigotes in 400 cells. The infection index, which is the percentage of infected macrophage multiplied by the average number of amastigotes per cell, was also estimated (21).

Briefly, the infection index was calculated by multiplying the percentage of infected macrophages by the average number of Leishmania per cell, which was determined by counting the cells under a fluorescent microscope.

### Parasite Quantitation by Real-Time PCR

Real-time PCR was used to quantify parasite burden in the spleen, 8 weeks after challenge. Initially, 1 × 10^6^ cells were isolated from spleen cell suspension and were stored at −20°C. Genomic DNA extraction was performed using a spin column-based nucleic acid purification kit (Parstous DNA Isolation Kit, Iran) according to the manufacturer's protocol. The specific primers were used to amplify a 75 bp fragment of the SODB1 gene of *L. major*. The primers' sequences previously designed, were 5'-TGGTG-GACATCATCAAGT-3' and 5'-AGAAGAAGTCGTGGTTGTA-3' (22). The reaction mixture contained 12.5 μL of master mix (Biofact, Korea), 1 μL of 2 pM of each primer, and 10 μL of the template DNA. The real-time PCR reaction was performed on a Rotor-Gene 6000 qPCR machine (Qiagen, Germany). The amplification times and temperatures were previously described (22). The non-template control (NTC) consisting of 10 μL water instead of the template DNA was used in each run. In order to plot a standard curve, a 10-fold serial dilution of *L. major* DNA, corresponding to 1 × 10^6^ parasites to 10^2^ parasites was prepared. The average cycle threshold (CT) of each dilution was plotted against the number of parasites. All assays were done in duplicates.

### Preparation of Soluble Leishmanial Antigen

The Soluble leishmanial antigen (SLA) was prepared from stationary phase *L. major*^*EGFP*^ *and ILL* promastigotes using repeated freezing and thawing (10 times), was followed by sonication (23). Briefly, 2 × 10^8^ promastigotes/mL were washed in 5 mL of cold sterile PBS three times. After 10 cycles of freezing and thawing, the suspension was sonicated three times 20 pulls with 40 W on ice then centrifuged at 5,000 × *g* for 20 min at 4°C. The supernatant containing SLA was collected and was stored at −70°C. The SLA protein concentration was determined by using the Bradford reaction (Cibzistfan, Iran).

### Anti-leishmania Antibody Level

Total anti-leishmanial antibody measurement was performed twice to confirm immunization. Mice were bled from retro-orbital sinus, 3 weeks after immunization (a day before challenge with *L. major*^*EGFP*^), and 8 weeks after challenge (the day before sacrificing). The mice sera were assayed using indirect ELISA for the presence of total IgG against soluble *Leishmania* antigen (SLA). Briefly, to determine anti-SLA IgG titer, ELISA plate (Greiner, Germany) was coated overnight (4°C) with 10 μg/mL of ILL SLA (SLA^ILL^) or *L. major* SLA (SLA^L.major^) in PBS (pH 7.2). The wells were washed with PBS containing 0.05% Tween-20. The plate was blocked using 200 μL of 1% bovine serum albumin (BSA) in PBS + 0.05% tween 20, for 1 h at room temperature. Then, 100 μL of diluted sera (1:40) was added to each well and followed by 1 h incubation at room temperature. For detection of specific total IgG, peroxidase-conjugated goat anti-mouse antibody (Santa Cruz Biotechnology, Inc., USA) was diluted 1:16000 and added to each well. After 1 h incubation at room temperature, 100 μL tetramethyl benzidine (TMB) (Razibiotech, Iran) was added. The enzymatic reaction was stopped with 100 μL 1 N H_2_SO_4_. Absorbance was recorded at 450 nm using an ELISA plate reader (Anthos 2020, Austria). The cut-off value was calculated as the mean of healthy controls' OD + 3 standards deviation. Each sample with higher OD than the cut-off value considered as immunized serum.

### Serum Levels of IgG1 and IgG2a Subclasses

To determine polarization of immune responses after immunization and after challenge, we evaluated IgG1 and IgG2a subclasses levels among studied groups. The evaluation was done using the commercial ELISA kits (Invitrogen, USA) and was performed according to the manufacturer's instructions.

### Cytokine Production Determination

Eight weeks' post-infection with *L. major*^*EGFP*^, mice were sacrificed, and single-cell suspensions of splenocytes were prepared in RPMI 1640 supplemented with 10% FBS and 1% Penstrep (Gibco, USA). Splenocytes were plated at 1 × 10^6^ cells/well (SPL, Korea) and re-stimulated by SLA^L.major^ (10 μg/mL). All tests were done in triplicate. Phytohemagglutinin (PHA) 2% was used as positive control. After 72 h incubation at 37°C in 5% CO_2_ and humid atmosphere, supernatants were harvested and the concentrations of IFN-γ, IL-4, and IL-10 were measured using commercial ELISA kits (respectively R&D, R&D, Biolegend, USA) according to the manufacturer's instructions.

### Nitric Oxide Griess Assay

Nitric oxide was measured in the splenocyte culture supernatant by Griess assay (21). Eight weeks after the challenge with *L. major*^*EGFP*^, mice were sacrificed and splenocytes (1 × 10^6^ cells/mL) were restimulated with SLA ^L.major^. The cultured cells (48 h) and supernatants (72 h) were collected to measure arginase activity and Nitrite concentration, respectively. The Griess assay determines nitrite concentration as a byproduct of the iNOS enzyme which indirectly indicates NO production. Briefly, Griess reagents including 1% sulfanilamide in 5% H_3_PO_4_, and NED solution including 0.1% naphthyl ethylene diamine (NED) dihydrochloride in distilled water (DW) were prepared. In a 96-well plate, 50 μL of sulfanilamide solution was added to 50 μL of sample and incubated 5 min in a dark place. Then, 50 μL of NED solution was added and incubated again for 5 min in a dark place. The absorbance was measured at 540 nm. Various concentrations (0–100 μM) of sodium nitrite in RPMI 1640 medium were used as standards to plot a standard curve.

### Measurement of Arginase Activity

The arginase activity was determined by measuring urea concentration as a byproduct of arginine decomposition using the micro-method (24). Briefly, splenocytes were seeded in 24-well plates (SPL, Korea) at a density of 1 × 10^6^ cells/well, re-stimulated by 10 μg/mL SLA ^*L*.*major*^ and incubated for 48 h at 37°C in 5% CO_2_ humid atmosphere. Then, cells from each well were harvested and mixed with 100 μL lysis buffer (0.02% Triton X100 and 2.5x protease inhibitor cocktail (Santa Cruz Biotechnology, Inc., USA) solution, pH 7.5) for 15 min with shaking. Afterward, the mix was centrifuged, and 25 μL of the supernatant was mixed with 25 μL of arginase activator solution (10 mM MnCl_2_, 50 mM Tris-HCl, PH = 7.5) followed by incubation at 56°C for 10 min. The activated lysate was incubated with 50 μL of 0.5 M L-arginine (pH = 9.7) at 37°C for 1 h. Reaction was stopped by adding 400 μL acid solution [H_2_SO_4_ (96%), H_3_PO_4_ (85%), H_2_O (1:3:7, v/v/v)] to each well. Various concentrations of urea (2–60 μg/mL) were prepared as standard, afterward, an acid solution (400 μL) was added to standards. Finally, 25 μL of 6% α-isonitrosopropiophenone (ISPF) (Sigma, USA) was added to samples and standards and incubated at 56°C for 7 min followed by 40 min at 100°C. In the end, all samples were transferred to a microplate, and the absorbance was measured at 540 nm. Arginase activity (mU) was calculated using the following formula:

(1) $$\begin{array}{l} \frac{1000\times 1000\times urea\;ug}{time\left(mins\right)\times sample\;volume\left(\mu L\right)\times urea\;molecular\;weight\left(ug\right)} \end{array}$$

The calculated arginase activity was divided into the total protein concentration measured using the bicinchoninic acid (BCA) assay kit (DNA biotech, Iran) and reported as mU/mg protein.

### Statistical Analysis

The Shapiro-Wilks test was used to detect departures from normality. Mann–Whitney *U*-test was used to compare the mean value of two groups; while for more than two groups Kruskal–Wallis one-way analysis of variance was done. To compare clinical scores data between four groups, repeated measure ANOVA test were used. *P* ≤ 0.05 was considered as statistically significant. All data were examined using SPSS (Ver. 24) software. In addition, all graphs were prepared using GraphPad Prism version 8.0.0 for Windows, (GraphPad Software, USA,). The data are presented as the mean ± standard deviation (*SD*).

## Results

### Immunization

**Figure 1:**
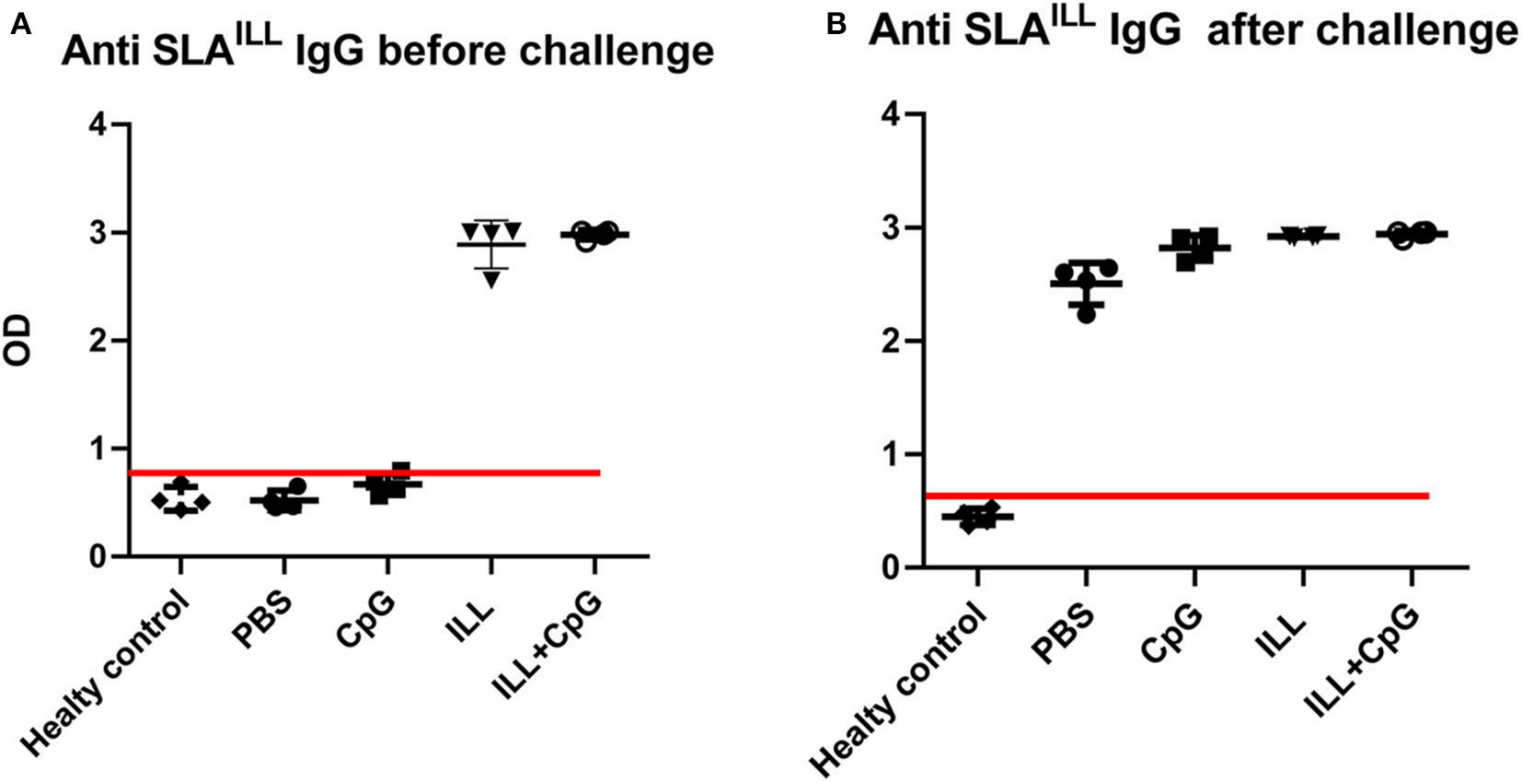
Determination of anti-SLA^ILL^ IgG antibody. **(A)** 3 weeks after immunization and **(B)** 8 weeks after the challenge with *L. major*^*EGFP*^, sera of all mice (*n* = 20) were collected. The level of the total anti-SLA^ILL^ IgG was determined using ELISA. Cut-off (red line) was determined using the mean of ODs of healthy controls + three standard deviations. Before challenge; mean + 3 *SD* = 0.866. After challenge; mean + 3 *SD* = 0.576. Each sample with higher OD than the cut-off value was considered as immunized serum. I.L.L (*Iranian Lizard Leishmania*), ILL+CpG (*Iranian Leishmania Lizard*+CpG). Data are shown as median with range.

We used live non-pathogenic parasite ILL mixed with CpG as an adjuvant, as a vaccine against *L. major*^*EGFP*^ infection in susceptible BALB/c mice. All mice groups were challenged 3 weeks after immunization with 2 × 10^5^ stationary phase *L. major*^*EGFP*^ promastigotes in their left, hind foot pad. Total specific IgG was measured in the serum samples of all groups including healthy mice. All immunized mice that received ILL parasites had specific IgG against SLA^ILL^ (Figure 1). Moreover, after challenge with *L. major*^*EGFP*^, the anti SLA^ILL^ IgG was detected in all groups expect healthy mice, due to antigenic similarities between ILL and *L. major*.

### Immunization With ILL+CpG Protected BALB/c Mice Against L. Major^EGFP^ Challenge

**Figure 2:**
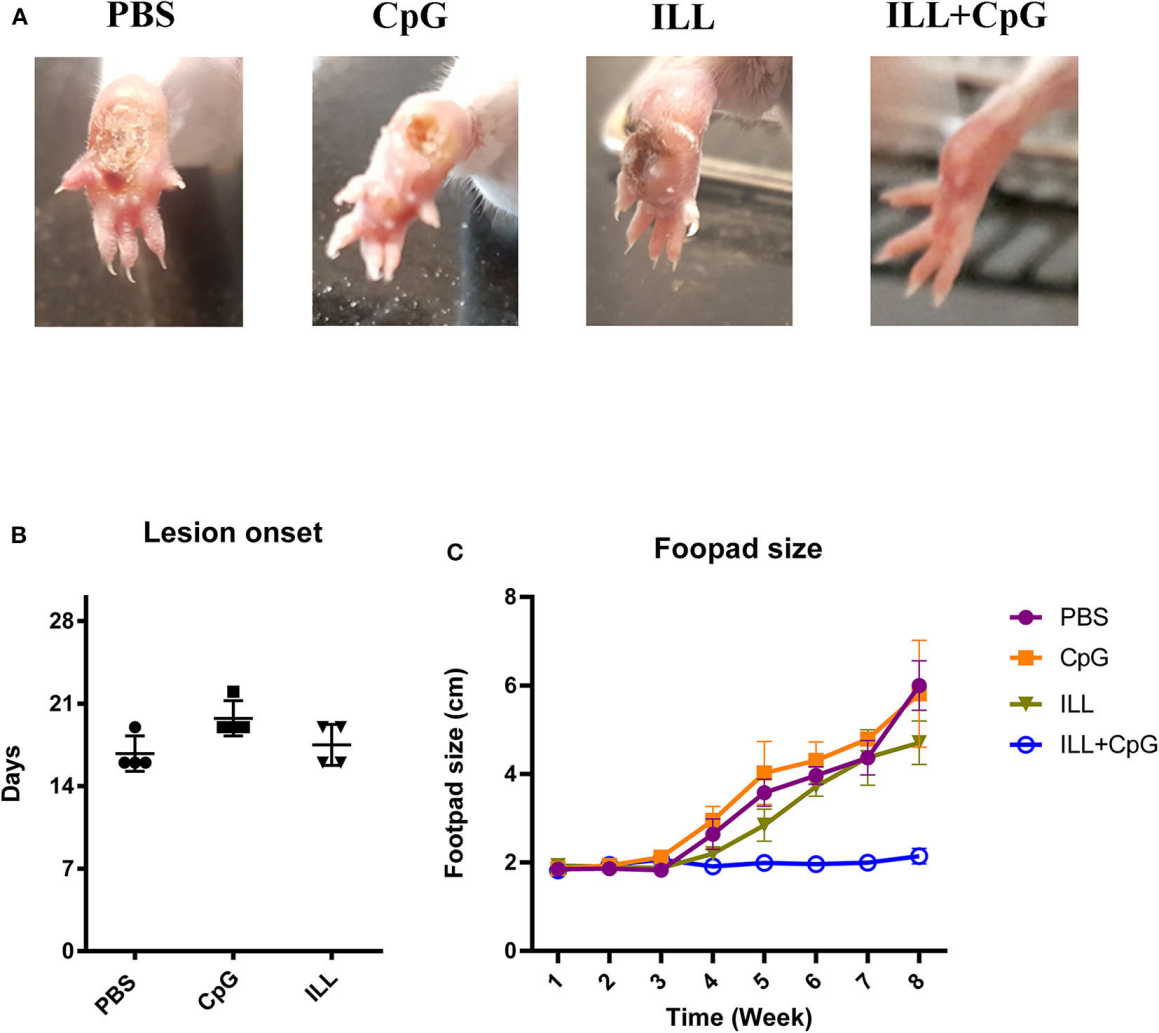
Footpad swelling and lesion onset. **(A)** The footpads of mice were monitored on a daily basis, after challenging with *L. major*^*EGFP*^. By the end of the 8th week, unlike the other groups, the ILL+CpG group showed no lesion at the injection site. **(B)** After challenge with *L. major*^*EGFP*^, footpad swelling was measured using a metric caliper, twice a week. As shown, the PBS group had the earliest lesion onset. **(C)** In addition, the footpads' diameter of the ILL+CpG group was at the basic level during the 8 weeks follow up. Data are shown as median with range. (*n* = 4 per group). I.L.L (*Iranian Lizard Leishmania*), ILL+CpG (*Iranian Leishmania Lizard*+CpG).

**Figure 3:**
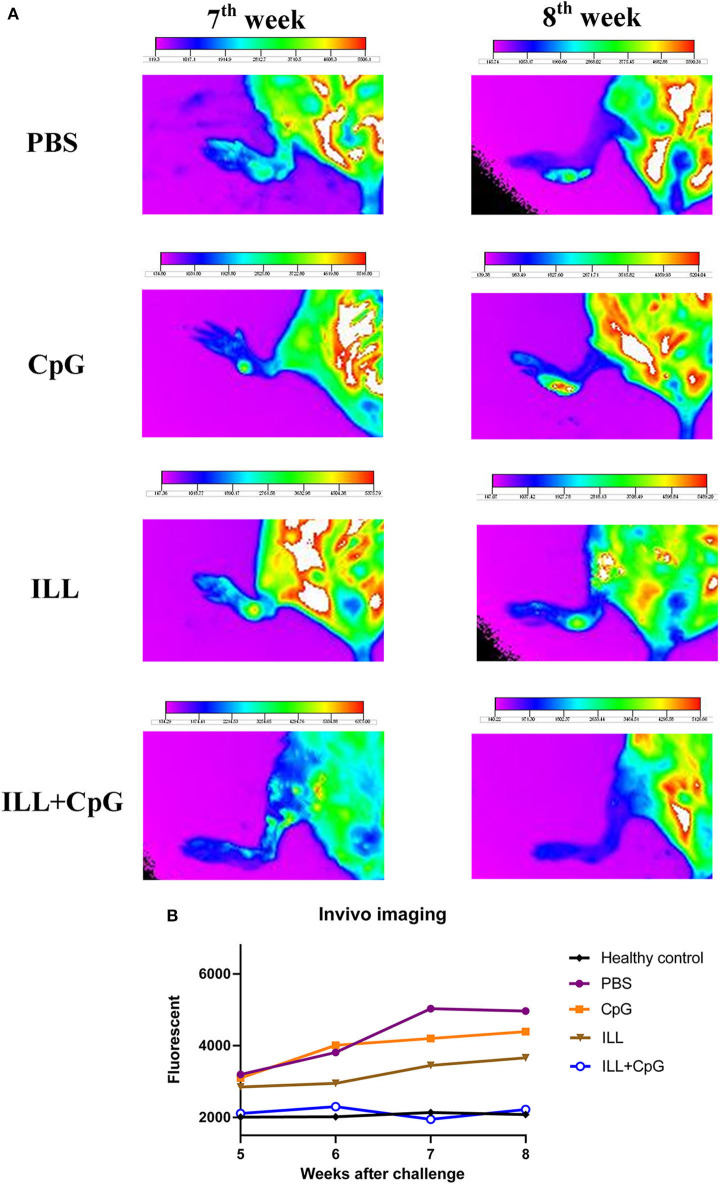
*In vivo* fluorescent imaging. **(A)** Fluorescent imaging was used to evaluate dynamic *L. major*^*EGFP*^ load at the site of infection (left footpad). Seven-weeks after the challenge with *L. major*^*EGFP*^, one mouse from each group was selected randomly and *in vivo*-imaging was performed. The fur of the leg was shaved and the mouse was anesthetized using Isoflurane 2% through the inhalation route, before imaging. The higher fluorescent intensity in the footpad indicates a higher parasitic load at the injection site. The arrows show the lesion site. Fluorescent intensity bar was drawn using Kodak Molecular Imaging software for each photo. **(B)** Considering the intensity bar, results showed that the ILL+CpG group had the lowest parasite load that was similar to the healthy group. Statistical analysis could not be performed because only one mouse per group was imaged. I.L.L (*Iranian Lizard Leishmania*), ILL+CpG (*Iranian Leishmania Lizard*+CpG).

The goal of vaccination is inducing a protective response to restrict the parasite number as well as limit the lesion formation. Therefore, monitoring the clinical manifestations of the disease is an important aspect of the evaluation of vaccine potency. In the present study, the ILL+CpG was the only group that was able to control *L.major*^*EGFP*^ infection regarding that only a moderate swelling without any open lesion was observed in the site of *L.major*^*EGFP*^ inoculation during 8 weeks of follow-up (Figure 2A). The results showed that in the ILL+CpG group, the lesion onset and footpad diameters (*P* = 0.021 from 4th week) were significantly less than those of the other groups (Figures 2B,C). Lesion onset could be considered as a criterion for the formation of a protective response. In addition to examining the clinical signs, protection against infection was determined using *in vivo* imaging. One mouse was randomly selected from each group and *in vivo*-imaging was performed in the 7 and 8th weeks after challenge with *L.major*^*EGFP*^. The higher fluorescence intensity in the footpad indicates a higher parasitic load at the inoculation site. Evaluation of fluorescence intensity revealed that parasitic burden increased in all groups except ILL+CpG group (Figures 3A,B). Given that a mouse was selected from each group for *in vivo*-imaging, the results of this evaluation were interpreted along with the results of other parasitic load measurements.

**Figure 4:**
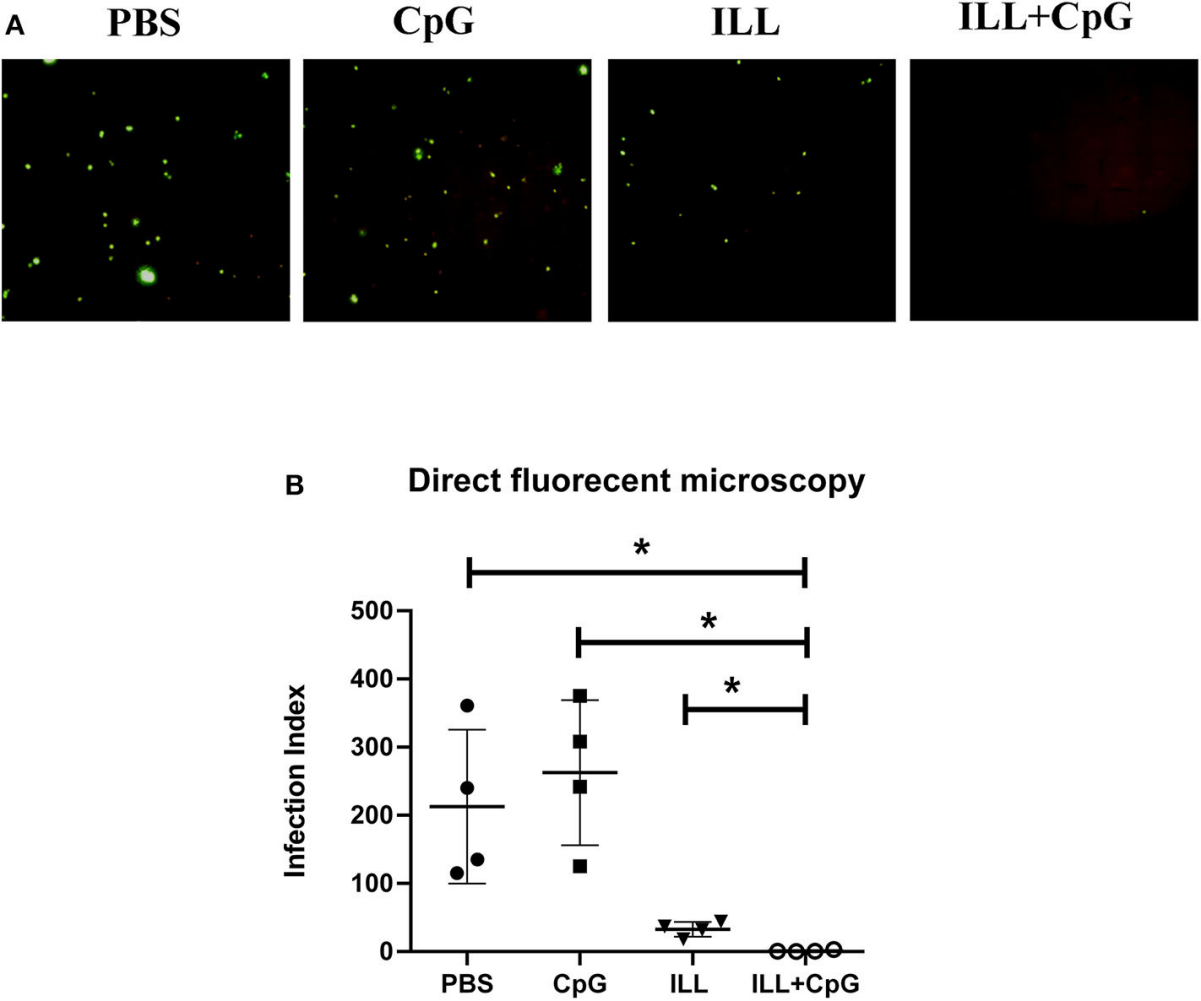
Parasite burden assay in popliteal lymph node using direct fluorescent microscopy. Eight weeks after challenge with *L. major*^*EGFP*^, mice were sacrificed and popliteal lymph nodes were isolated. **(A)** The cell suspension was prepared. The parasites expressing green fluorescent protein (GFP) were imaged using a fluorescent microscope. **(B)** To compare parasite burden between groups, the infection index was calculated by multiplying the percentage of infected macrophages by the average number of parasites per cell. The infection index was significantly lower in immunized groups than controls. To compare the means, the Mann–Whitney *U*-test was used. (^*^ *P* ≤ 0.050). Data are shown as mean ± *SD*.

In addition, in order to determine the parasitic load in the lymph node, direct fluorescent microscopy was performed and the infection index was calculated (Figure 4A). Results showed that mice immunized with ILL+CpG had a significant reduction in infection indexes in comparison to the PBS control group (*P* = 0.021, Figure 4B).

**Figure 5:**
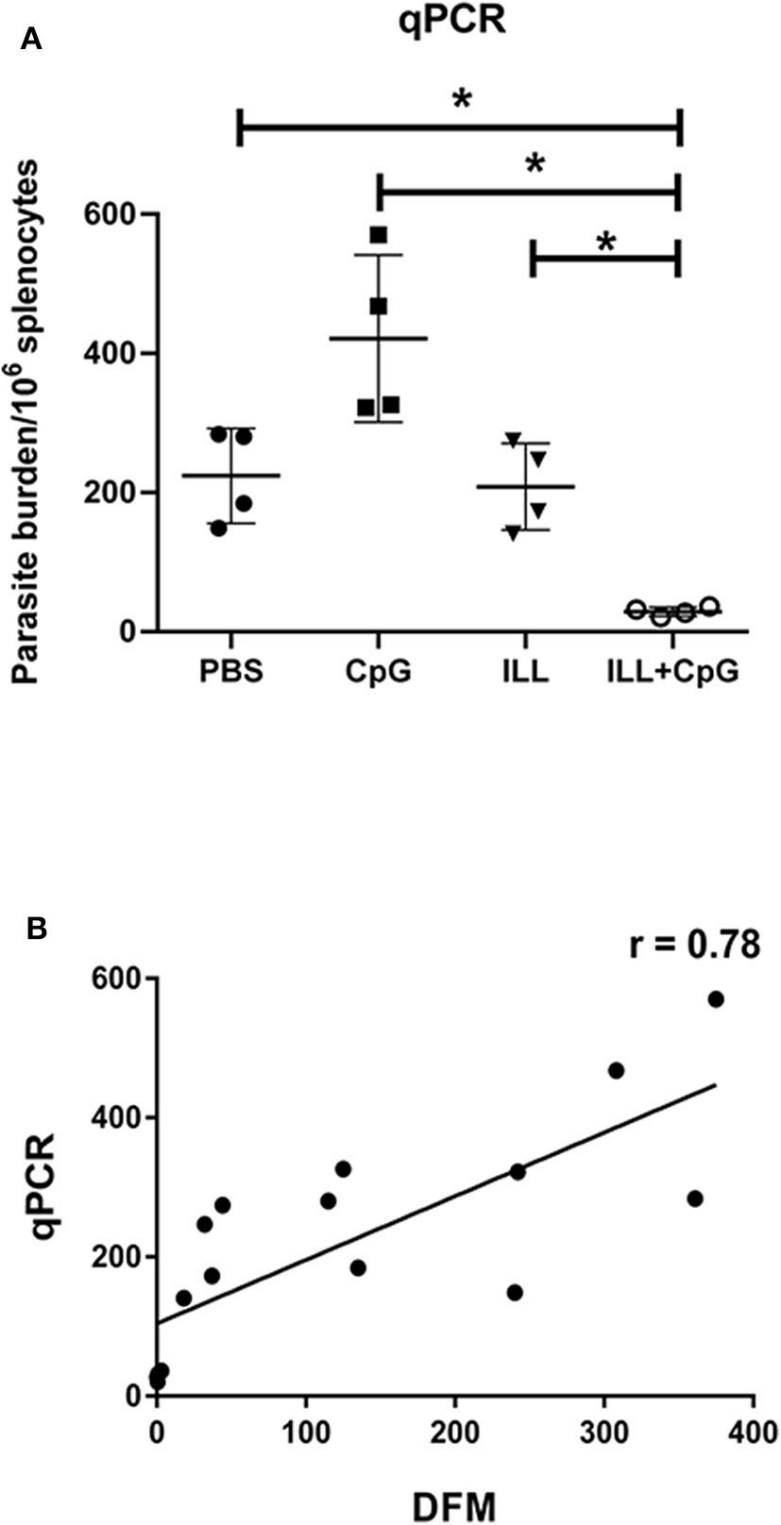
Parasite burden analysis in splenocytes using quantitative real-time PCR assay. **(A)** Eight weeks after challenge all mice (*n* = 4 per group) were sacrificed and spleens were isolated. Genomic DNA was extracted and the amount of *Leishmania* DNA was quantified via amplifying the SODB1 gene of *L. major* using real-time PCR assay. In the ILL+CpG group compared to other groups, parasite burden was significantly decreased. **(B)** In order to determine the correlation coefficient between the direct fluorescent microscopy (DFM) in popliteal lymph node cells and qPCR in splenocytes, statistical analysis was performed. Spearman's correlation coefficient of the parasite burden between the two techniques was 0.78 and was statistically significant (*P* = 0.0004). The asterisks indicate the significant differences between groups as determined by a Mann–Whitney *U*-test (*P ≤ 0.05). Data are shown as mean ± *SD*.

Moreover, 8 weeks after the challenge, the amount of parasite dissemination to the spleen was quantified using real-time PCR. As expected, immunization with ILL+CpG significantly reduced parasite burden in comparison to PBS and CpG groups (*P* = 0.021, Figure 5A). There is a strong positive correlation between the parasite load measured using the direct fluorescent microscopy on samples taken from draining lymph node and qPCR assay done on splenocytes specimen (*r* = 0.78, *P* = 0.0004, Figure 5B).

### Immunization With Live ILL Plus CpG Increased IFN-γ and Reduced IL-4 Production

**Figure 6:**
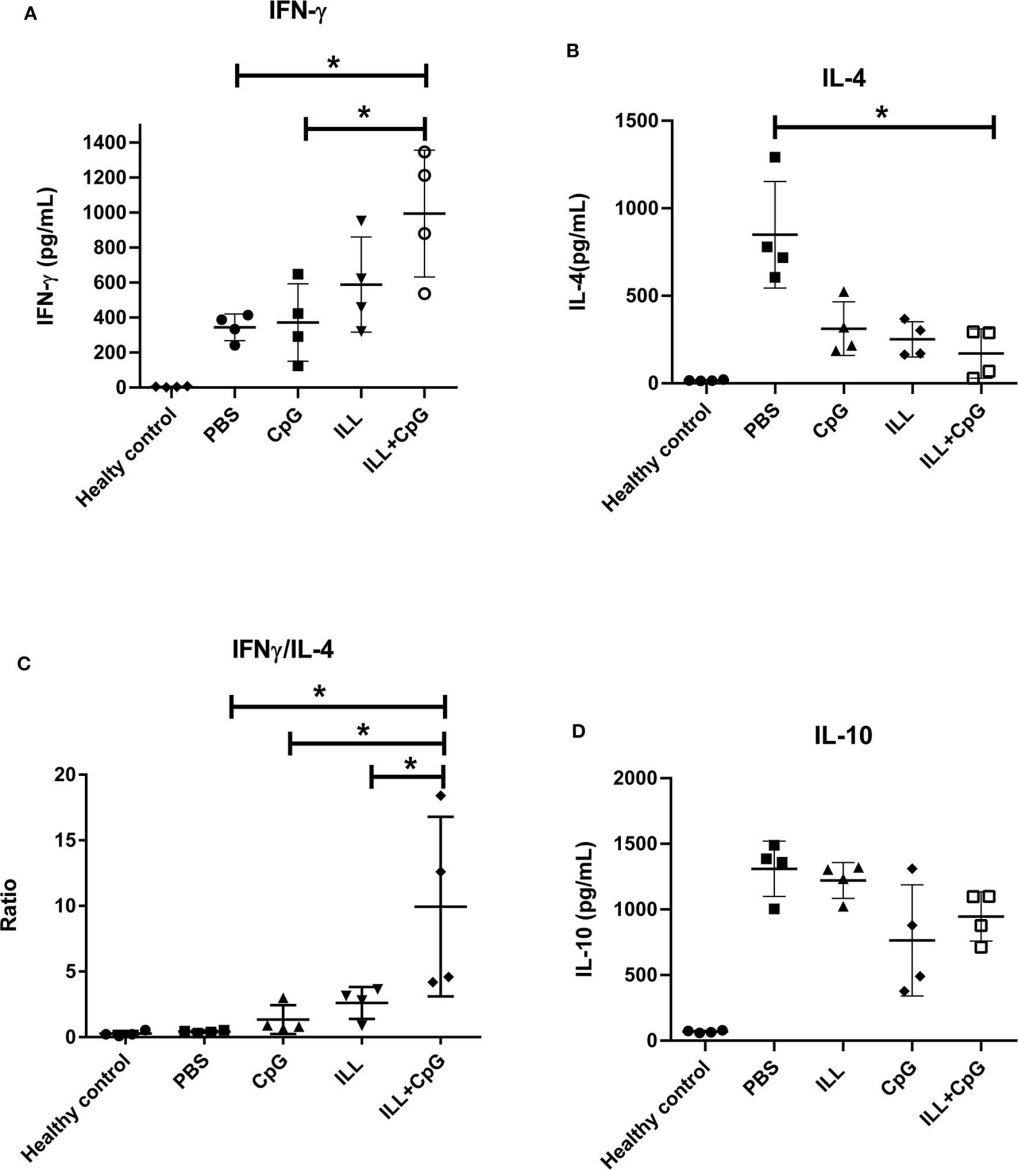
The levels of IFN-γ, IL-4, and IL-10 cytokines in splenocytes culture. Eight weeks after challenge all mice including healthy mice (*n* = 4 per group) were sacrificed and splenocytes were re-stimulated with SLA^L.major^ and PHA. After 72 h, supernatants were collected and cytokines were measured using an ELISA assay. **(A)** IFN-γ level was significantly higher in the ILL+CpG group. **(B)** However, the level of IL-4 was significantly higher in the PBS group **(C)** IFN-γ/IL-4 ratio shows a T_H_1 mediated response and in the ILL+CpG group was higher than controls. **(D)** IL-10 levels were not statistically significant differences between studied groups. The asterisks indicate the significant differences between groups as determined by a Mann–Whitney *U*-test (*P ≤ 0.05). Data are shown as mean ± *SD*. I.L.L (*Iranian Lizard Leishmania*), ILL+CpG (*Iranian Leishmania Lizard*+CpG).

The protection conferred by ILL plus CpG could be due to the potency of CpG-ODN to induce a T_H_1 immune response. Therefore, we measured IFN-γ (as the main T_H_1 cytokine), IL-4 (as the T_H_2 prototype cytokine), and IL-10 (as an immunomodulatory cytokine) in the supernatant of splenocytes re-stimulated with SLA^L.major^. As shown in Figure 6A, the ILL+CpG group produced a substantially higher level of IFN-γ in comparison to the PBS control group (*P* = 0.021). The ILL+CpG group also released a lower concentration of IL-4 than the PBS group (*P* = 0.021) (Figure 6B). The post-challenge IFN-γ/IL-4 ratio in response to stimulation with SLA^L.major^ is a valuable indicator of vaccine potency. Results showed that IFN-γ/IL-4 ratio was higher in the ILL+CpG group when compared to each of the other groups (*P* = 0.021 in comparison to PBS) (Figure 6C). We also measured the secretion of IL-10 as a major anti-inflammatory cytokine. The results showed that the IL-10 level in the ILL+CpG group was similar to the other groups (*P* > 0.05) (Figure 6D).

**Figure 7:**
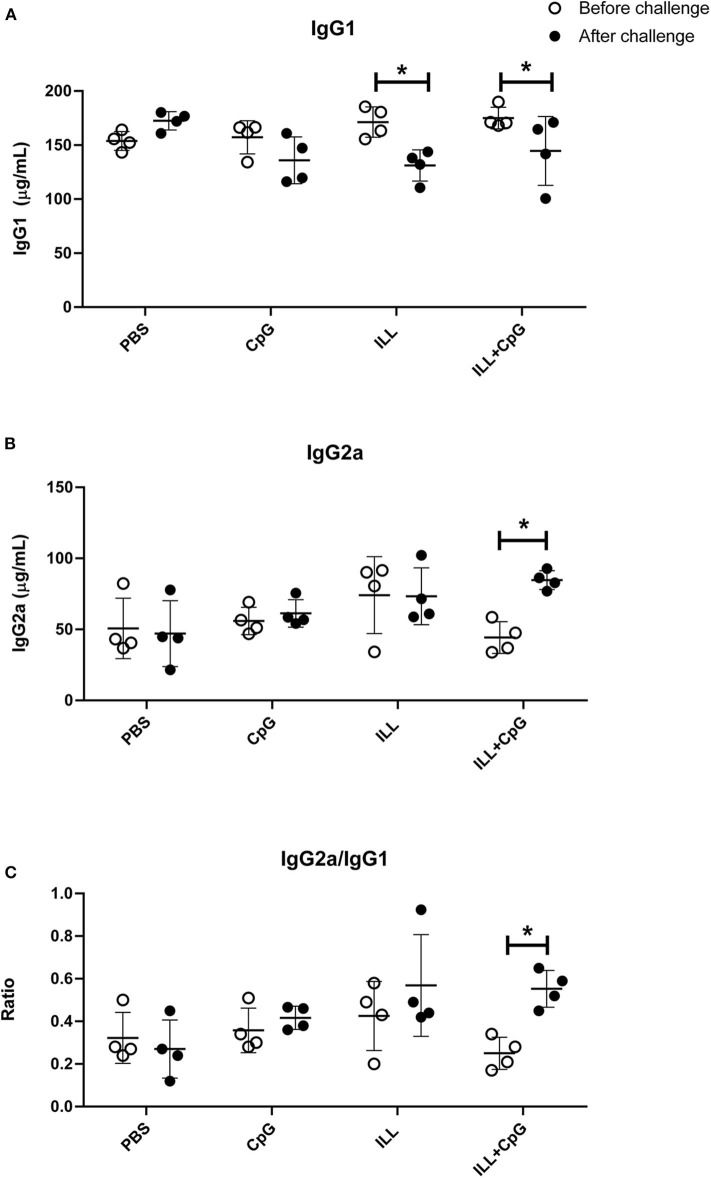
Determination of humoral immune response in mice. Total IgG1 and total IgG2a levels were measured two times using ELISA. Mice were bled 3 and 8 weeks after challenge with *L. major*^*EGFP*^. **(A)** IgG1 levels before and after challenge. The level of IgG1 significantly decreased in immunized mice (ILL and ILL+CpG groups), after the challenge. **(B)** IgG2a levels before and after the challenge. After the challenge, the level of IgG2a was increased significantly only in the ILL+CpG group. **(C)** IgG2a/IgG1 ratio levels before and after challenge. The IgG2a/IgG1 ratios were increased in ILL+CpG after challenge. Mann–Whitney *U*-test was performed to compare antibody levels before and after the challenge of each group and significant differences are shown with asterisks (*P ≤ 0.05). Data are shown as mean ± *SD*. I.L.L (*Iranian Lizard Leishmania*), ILL+CpG (*Iranian Leishmania Lizard*+CpG).

We also determined the humoral response through the assessment of IgG subclasses. It has been shown that IgG2a antibody induction is correlated with IFN-γ production and T_H_1 responses, while IgG1 production is associated to the T_H_2 immune profile (25). The total IgG2a and the total IgG1 levels were determined in mice sera, 3 weeks after immunization, and 8 weeks after challenge. The results showed that before the challenge, there was no difference in the total IgG1 and the total IgG2a levels between the groups (Figures 7A,B). However, after the challenge, the PBS group had higher total IgG1 antibody level compared to immunized groups (*P* = 0.001). Moreover, there was a significant difference in IgG2a level between the immunized groups and the PBS group after challenge (*P* = 0.021, Figure 7B). Additionally, the results showed that 8 weeks after the challenge, in the ILL+CpG group, the IgG1 level was decreased while the IgG2a level was increased (*P* = 0.021 and *P* = 0.021, respectively). Therefore, IgG2a/IgG1 ratios were increased in ILL+CpG after the challenge in comparison with before the challenge in this group (Figure 7C, *P* = 0.021).

### Immunization With ILL+CpG Reduced Arginase Activity

**Figure 8:**
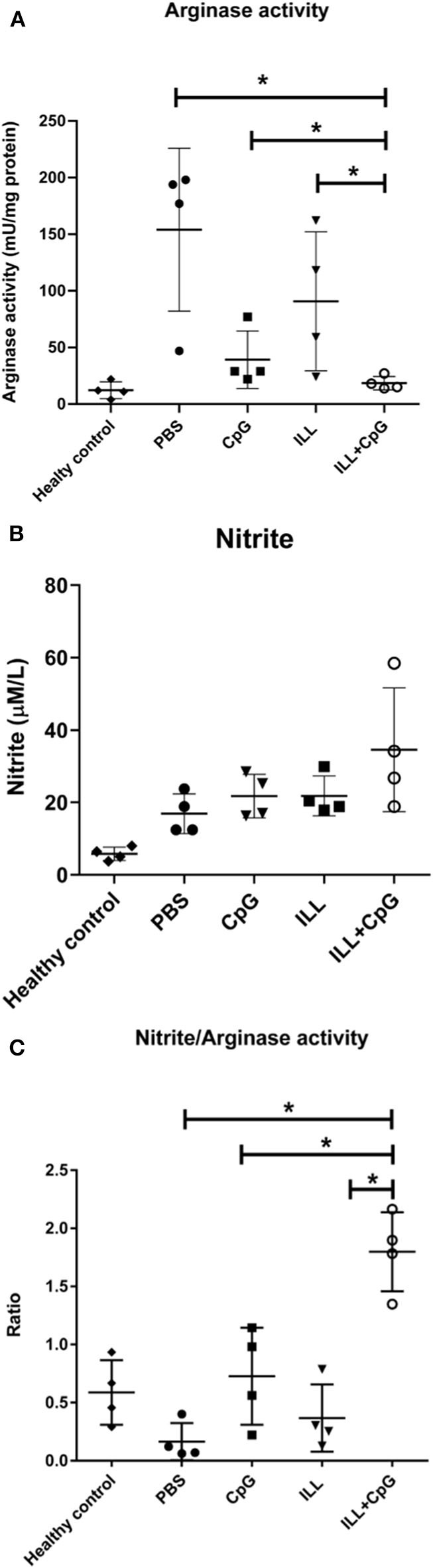
Arginase and iNOS activity in immunized and control groups. Eight weeks after challenge with *L. major*^*EGFP*^, mice were sacrificed and splenocytes (1 × 10^6^ cells/mL) were re-stimulated with SLA^L.major^. To measure arginase activity, the cultured cells were collected after 48 h. In addition, to evaluate the nitrite concentration the supernatant of the culture was used. **(A)** Arginase activity was significantly lower in the ILL+CpG group in comparison to controls. **(B)** There was no significant difference between nitrite concentration in the studied groups. **(C)** The nitrite concentration/Arginase activity ratio showed immune protective responses. The ratio was significantly higher in the ILL+CpG group than in other groups. *n* = 4 per group. The means were compared with a Mann–Whitney *U*-test and significant differences are shown with asterisks (*P ≤ 0.05). Data are shown as mean ± *SD*. I.L.L (*Iranian Lizard Leishmania*), ILL+CpG (*Iranian Leishmania Lizard*+CpG).

Arginase and iNOS are two inducible enzymes that convert arginine as a substrate to active mediators which crucially affect the *Leishmania* infection outcomes. In the current study, the arginase activity and nitrite concentrations were measured in the culture of splenocytes, 8 weeks after the challenge. Regarding the instability of NO, determination of the nitrite concentration which is produced after NO refraction considered as an indicator for iNOS activity and NO level. The results showed that the ILL+CpG group had a lower arginase activity level (4.75 ± 1.7 mU/mg protein) than the PBS group (154 ± 71.91 mU/mg protein, *P* = 0.021, Figure 8A). However, no significant difference was observed among the immunized groups and control groups in the levels of nitrite (*P* > 0.05, Figure 8B). Nevertheless, the nitrite/arginase activity ratio was significantly higher in the ILL+CpG group mice compared to the controls (*P* = 0.021, Figure 8C). These findings confirm that immunization using ILL+CpG is able to limit *L. major* growth by inducing anti-leishmanial responses in host macrophages.

## Discussion

In the present study, *Iranian Lizard Leishmania*, mixed with CpG-ODN, was used to induce protective immunity against *L. major*^EGFP^ infection in BALB/c mice. The results showed that subcutaneous inoculation of the live vaccine resulted in a potent protective immunity against *L. major*^EGFP^ infection, which was revealed by the absence of open lesions in the footpad of mice challenged with *L. major*^EGFP^. The observed protection was associated with mild swelling in the foot-pad and low parasitic load in spleen and draining lymph nodes. The results revealed that the protection was mediated by increased IFN-γ production, decreased IL-4 secretion, as well as decreased arginase activity.

*Leishmania* produces zoonotic infections. For instance, members of the subgenus *L. Sauroleishmania* can be isolated from reptiles. *Leishmania tarantolae* was detected in the gecko *Tarenola annularis* in Sudan (26). *Iranian lizard Leishmania* was isolated from *Agama caucasica microlepis* captured in Shahrod province in Iran (27). Ultrastructure study using electron microscopy as well as cloning and the enzymatic assay of pteridine reductase-1 showed that *ILL* differs from other *lizard Leishmania* promastigotes that previously were isolated in other countries (28, 29). Breton et al. reported that *L. tarentolae* does not persist more than 1 month in infected BALB/c mice and was not able to generate any manifestation of leishmaniasis (6). Therefore, it seems that the main obstacle to using *L. tarentolae* as a live vaccine is its limited infectivity. To overcome this barrier, finding more virulent leishmania strain was considered. Previous studies have shown that not all *lizard Leishmania* are non-pathogenic to humans. For example, *L. adleri* is able to produce a lesion in humans, and some strains of *L. tarentolae* (LEM 125) could cause a transient infection (7). Our findings showed that in ILL infected mice a transient lesion was detected in the inoculation site and therefore we believed that it sustained longer durability in the body that would elicit more effective immune responses.

Although we did not survey the persistent of ILL in studied groups, our findings showed that in ILL-infected mice a transient lesion was formed at the site of inoculation, therefore we assumed that ILL sustained longer durability in the body than *L. tarantolae*; then it may able to elicit more effective immune responses, which is necessary for long term immunity. Undeniably, the probable higher infectivity increases the concerns about the vaccine biosafety for human use. All of this suggests that further studies are needed.

The results of the present study showed that ILL mixed with CpG-ODN conferred a strong protective response resulting in no open lesion at the site of the challenge with *L. major*^EGFP^. Mendez et al. showed that using CpG with live *L. major* reduced the size and duration of active lesions very effectively (14). Indeed, inoculation of live *L. major* mixed with CpG induced a T_H_1 immune response and long-term immunity (28). Similarly, previous findings revealed that the protection that was achieved by immunizing BALB/c mice with live *L. tarentolae* secreting the sand fly salivary antigen in the presence of CpG-ODN was mediated by T_H_1 responses (8). In the present study, immunity induced by ILL+CpG reduced the parasite load in mice. By using *in vivo* imaging, it was observed that the ILL+CpG group had the lowest fluorescent intensity in the challenge site; in addition, the parasite indexes measured in popliteal lymph nodes using the DFM method showed that the ILL+CpG group had ~190-fold lower parasite burden than the other studied groups. Moreover, the qPCR assay that was used to determine parasite load in spleen cells revealed that the immunized group had about 87% lower parasite burden. Similarly, Breton et al. observed that after vaccination with *L. traentolae* the spleen parasite burden measured by luciferase activity was reduced by 85% (6). Moreover, in another study it was reported that the combination of *L. traentolae* and CpG could reduce the lymph node parasite load (8). It should be added that Real-time PCR and DFM present a snapshot view of parasite burden, so, to get a dynamic view, it is necessary to use live animals. The *in vivo* imaging was performed from the 7th week after challenge with *L.major*^EGFP^ to get a more comprehensive results.

As has been revealed previously, a milieu of T_H_1 type cytokines leads to control of *Leishmania* infection while a T_H_2 milieu results in the disease progression. Indeed, T_H_2 milieu supports a humoral response, which has little or no effect on intracellular parasites. T_H_1 cytokines, mainly IFN-γ, stimulate macrophages to kill *Leishmania* parasites (30). In the current study, ILL+CpG group produced higher quantities of IFN-γ than controls. On the other hand, IL-4 levels were significantly decreased in the ILL+CpG group compared to the PBS control group. Moreover, the highest IFN-γ/IL-4 ratio belonged to the ILL+CpG group while the lowest ratio was observed in the PBS group, which was consistent with the clinical findings.

The results of the current study showed that the immunized mice produced a similar level of IL-10 in comparison with other groups. IL-10 is a principal regulatory cytokine that suppresses the activity of macrophages and T_H_1 cells that are essential for parasite control (31). On the other hand, IL-10 plays a pivotal role in the acceleration of the wound healing process (31). The main sources of IL-10 are regulatory T cells and T_H_2 cells. Interestingly, IL-10 can also be produced by T_H_1 and macrophages that secrete IFN-γ (31). Hence, T_H_1 cells and macrophages auto-regulate themselves by IL-10 production. Therefore, a balanced IFN-γ/IL-10 axis could support the clearance of the parasite by macrophages, as well as control the deleterious side-effect of exaggerated inflammation (32). The results of the present study showed that the high production level of IFN-γ in the ILL+CpG group was accompanied by significant IL10 production in this group.

One of the pathways play an important role in the fate of *Leishmania* infection is the arginine metabolic pathway. Arginase catabolizes L-arginine into urea and ornithine. The latter is further catabolized to polyamines that are required for *Leishmania* parasite growth. By contrast, inducible nitric oxide synthase (iNOS) oxidizes L-arginine in a two steps process to nitric oxide (NO), which is a metabolite responsible for the *Leishmania* parasite clearance. Both enzymes use L-arginine as a shared substrate, hence the arginase pathway inhibits the iNOS pathway, and vice versa (33). In the present study, there was no increase in nitrite level in the ILL+CpG group compared to other groups except healthy mice. On the other hand, the lowest arginase activity was observed in the ILL+CpG group, while the highest activity was observed in the PBS controls. The study by Virginia et al. showed that the *L. major* growth increased in macrophages *in vitro* by the induction of arginase-I and that arginase inhibition reduced the number of parasites and delayed lesion onset in infected BALB/c mice (34). By contrast, in resistant mice such as C57BL/6, a protective response caused the arginase activity to be maintained at the baseline level, which was associated with the control of lesions (34).

In the previous studies, it has been well-established that T_H_1 cellular responses are associated with a high level of IFN-γ production and increased level of IgG2a antibody subclass (35), whereas T_H_2 response augments the production of IL-4 as well as IgG1 subclass (36). In the present study, a considerable decrease in the total IgG1 concentration, along with a significant increase in the total IgG2a level after the challenge was observed in the immunized group. The observed alteration in antibody levels was reflected by a high IgG2a/IgG1 ratio, indicates the induction of a more pronounced T_H_1 response. However, since the total IgG subclasses were determined, the determination of specific anti-leishmanial antibodies may prepare more reliable and coordinating results with the cytokines assays.

## Conclusion

The results demonstrate that ILL+CpG vaccination induced a strong protective response, suggest that *ILL* with an appropriate adjuvant could be a suitable choice for safe leishmanization. Although the observed protective immune response is a short-term response, it could be a step forward to other studies that will be done for inducing long-term immunity.

## Data Availability Statement

Data are available from the authors upon reasonable request and with permission of the research deputy of Shahid Beheshti University of Medical Sciences. Requests to access the datasets should be directed to fyeganeh@gmail.com.

## Ethics Statement

The animal study was reviewed and approved by Institutional Animal Care and Research Advisory Committee of the Shahid Beheshti University of Medical Sciences, Tehran, Iran (IR.SBMU.MSP.REC. 1396.743).

## Author Contributions

FY devised the project, the main conceptual ideas, and proof outline. NK worked out almost all of the technical details with help from MN and performed the statistical analysis for the suggested experiment. MH proposed the methods in discussions with FY and edited the outline. All authors contributed to the article and approved the submitted version.

## Conflict of Interest

The authors declare that the research was conducted in the absence of any commercial or financial relationships that could be construed as a potential conflict of interest.

## Footnotes

^1^Cellular and Molecular Biology Research Center, Shahid Beheshti University of Medical Sciences, Tehran, Iran.
